# Tissue and Stage-Specific Distribution of *Wolbachia* in *Brugia malayi*

**DOI:** 10.1371/journal.pntd.0001174

**Published:** 2011-05-24

**Authors:** Kerstin Fischer, Wandy L. Beatty, Daojun Jiang, Gary J. Weil, Peter U. Fischer

**Affiliations:** 1 Infectious Diseases Division, Department of Internal Medicine, Washington University School of Medicine, St. Louis, Missouri, United States of America; 2 Department of Molecular Microbiology, Washington University School of Medicine, St. Louis, Missouri, United States of America; George Washington University Medical Center, United States of America

## Abstract

**Background:**

Most filarial parasite species contain *Wolbachia*, obligatory bacterial endosymbionts that are crucial for filarial development and reproduction. They are targets for alternative chemotherapy, but their role in the biology of filarial nematodes is not well understood. Light microscopy provides important information on morphology, localization and potential function of these bacteria. Surprisingly, immunohistology and *in situ* hybridization techniques have not been widely used to monitor *Wolbachia* distribution during the filarial life cycle.

**Methods/Principal Findings:**

A monoclonal antibody directed against *Wolbachia* surface protein and *in situ* hybridization targeting *Wolbachia* 16S rRNA were used to monitor *Wolbachia* during the life cycle of *B. malayi*. In microfilariae and vector stage larvae only a few cells contain *Wolbachia*. In contrast, large numbers of *Wolbachia* were detected in the lateral chords of L4 larvae, but no endobacteria were detected in the genital primordium. In young adult worms (5 weeks p.i.), a massive expansion of *Wolbachia* was observed in the lateral chords adjacent to ovaries or testis, but no endobacteria were detected in the growth zone of the ovaries, uterus, the growth zone of the testis or the *vas deferens*. Confocal laser scanning and transmission electron microscopy showed that numerous *Wolbachia* are aligned towards the developing ovaries and single endobacteria were detected in the germline. In inseminated females (8 weeks p.i.) *Wolbachia* were observed in the ovaries, embryos and in decreasing numbers in the lateral chords. In young males *Wolbachia* were found in distinct zones of the testis and in large numbers in the lateral chords in the vicinity of testicular tissue but never in mature spermatids or spermatozoa.

**Conclusions:**

Immunohistology and *in situ* hybridization show distinct tissue and stage specific distribution patterns for *Wolbachia* in *B. malayi*. Extensive multiplication of *Wolbachia* occurs in the lateral chords of L4 and young adults adjacent to germline cells.

## Author Summary

Most filarial nematodes contain *Wolbachia* endobacteria that are essential for development and reproduction. An antibody against a *Wolbachia* surface protein was used to monitor the distribution of endobacteria during the *B. malayi* life cycle. *In situ* hybridization with probes binding to *Wolbachia* 16S rRNA were used to confirm results. Only a few cells contain *Wolbachia* in microfilariae and vector stage larvae; this suggests that the bacteria need to be maintained, but may have limited importance for these stages. Large numbers of *Wolbachia* were detected in the lateral chords of L4 larvae and of young adult worms, but not in the developing reproductive tissue. Confocal laser scanning and transmission electron microscopy showed that *Wolbachia* are aligned towards the developing germline. It can be hypothesized that *Wolbachia* invade developing ovaries from the lateral chords. In inseminated females, *Wolbachia* were detected in the ovaries and embryos. In young males, *Wolbachia* were found in parts of the testis and in the lateral chords in the vicinity of testicular tissue but never in mature spermatids or spermatozoa. The process of overcoming tissue boundaries to ensure transovarial transmission of *Wolbachia* could be an Achilles heel in the life cycle of *B. malayi*.

## Introduction

Filarial parasites infect more than 150 million people in tropical and subtropical countries and are responsible for important tropical diseases such as lymphatic filariasis (elephantiasis) and onchocerciasis (river blindness). Other filarial species are important veterinary pathogens (e.g. *Dirofilaria immitis*, the dog heartworm). Treatment of filarial infections in humans and animals is suboptimal, because available drugs do not efficiently kill adult worms. Most filarial species live in obligatory symbiosis with intracellular *Wolbachia* α-proteobacteria. *Wolbachia* are also present in many insect species, and they are among the most widely distributed bacteria that infect invertebrates. *Wolbachia* endosymbionts are necessary for development and reproduction of filarial nematodes, and they have been validated as a target for chemotherapy [1]. Tetracycline class antibiotics are active against *Wolbachia*, and depletion of endobacteria blocks reproduction and eventually kills adult worms in some filarial species [2], [3].

While *Wolbachia* DNA can be detected and quantified by PCR, microscopy provides important information on morphology and localization of bacteria in parasite tissues. Immunohistochemistry has been used for years to visualize *Wolbachia* in filarial worms, particularly in *Onchocerca volvulus* [2]. *Brugia malayi* is the only human filarial parasite that can be maintained in laboratory animals and for which all life cycle stages are relatively easily accessible. The population dynamics of *Wolbachia* during the development of *B. malayi* has been studied by quantitative PCR; for example, the number of *Wolbachia* exponentially increases soon after infection of the vertebrate host [4]. Recent studies have shown that *Wolbachia* are unevenly distributed in intrauterine embryos and that the bacteria are not always detected in germline precursor cells [5]. However, data on the histological distribution of *Wolbachia* during later development of *B. malayi* are scarce. While it is known that *Wolbachia* are present in developing embryos, the mechanism of this vertical transmission is poorly understood.

*In situ* hybridization has been used to study gene expression in filarial parasites such as *B. malayi* [6] and to detect *Wolbachia* in insects [7], [8], but it has not been used before to detect *Wolbachia* in filarial worms. In this paper, we have used optimized immunohistology, *in situ* hybridization, and transmission electron microscopy to systematically describe the distribution, the relative number and morphology of *Wolbachia* in different life stages and tissues of *B. malayi*. This work led to an interesting new hypothesis on the localization and migration of *Wolbachia* during development of filarial worms.

## Materials and Methods

### Parasite material

*B. malayi* worms were recovered from intraperitonial ( i.p.) infected jirds, 2, 5, 8 and 12 wks post infection (p.i.) as previously described [9]. *Aedes aegypti* mosquitoes containing different larval stages of *B. malayi* were available from a previous study. Parasite material was fixed either in 80% ethanol for immunohistology or in 4% buffered formalin for immunohistology or *in situ* hybridization. At least five blocks with four or more *B. malayi* worms each were examined for each time point. An extensive overview about the studied material and the methods performed is provided in a supplementary table (Table S1). Up to twenty serial sections of the same block were used for comparative studies of different staining procedures. For some blocks (especially those containing young adult worms) more than 60 sections (5 µm) were cut, but only a selection of sections was examined. For the ultrastructural analysis, 18 worms (39 and 56 days p.i., Table S1) were fixed in 2% paraformaldehyde/2.5% glutaraldehyde (Polysciences Inc., Warrington, PA, USA) in 100 mM phosphate buffer, pH 7.2 for 1 hr at room temperature.

### Antibodies

A monoclonal antibody directed against the *B. malayi Wolbachia* surface protein (mab *Bm* WSP) was purified from culture supernatants kindly provided by Dr. Patrick J. Lammie, Atlanta [10]. Briefly, hybridoma supernatant was incubated overnight at 4°C with ammonium sulfate, pelleted, resuspended in water and dialyzed extensively against phosphate buffered saline. The antibody solution was concentrated to 5% of the original volume using Centricon Plus-20 columns (Millipore, Billerica, MA, USA) and the protein content was determined. A stock mab solution of 10 mg protein per ml was used to test dilution series of 1∶10 up to 1∶500. The best signal to background relationship was observed at a dilution of 1∶100, and this dilution was used for all further experiments.

### Immunohistology

The alkaline phosphatase anti-alkaline phosphatase (APAAP) technique was applied for immunostaining according to the recommendations of the manufacturer (Dako, Carpinteria, CA, USA) and as described earlier [11]. TBS with 1% albumin was used as negative control. Rabbit-anti mouse IgG (1∶25; Dako) was applied as secondary antibody and was bound to the APAAP complex. As substrate for alkaline phosphatase the chromogen Fast red TR salt (Sigma) was used and hematoxylin (Merck, Darmstadt, Germany) served as the counter-stain. Sections were examined using an Olympus-BX40 microscope (Olympus, Tokyo, Japan) and photographed with an Olympus DP70 microscope digital camera. For some fluorescent analysis wheat germ agglutinin (WGA 633, Invitrogen, Carlsbad, CA, USA) was used as membrane stain at 200 µg/ml for 10 minutes prior to mounting.

FITC conjugated anti-mouse IgG (1∶300; Sigma) was used as a secondary antibody for confocal laser scanning microscopy (LSM). Sections were examined with a Zeiss LSM 510 META (Zeiss, Jena, Germany) confocal laser scanning microcope equipped with a plan-apochromat 63× oil objective with an argon or helium/neon laser for excitation at 488 nm or 633 nm, respectively. Confocal Z slices of 0.8 µm were obtained using Zeiss LSM software. The Velocity program version 5.4.2 (Improvision, Lexington, MA, USA) was used for high resolution interactive 3D rendering. Sections were also examined using a wide field fluorescence microscope (WFFM, Zeiss Axioskop 2 MOT Plus) with plan-apochromat 100× oil, 63× or 40× objectives. Wide field fluorescence microscopy and LSM were performed at the Washington University Molecular Microbiology Imaging Facility (http://micro.imaging.wustl.edu/).

### rRNA probe *in situ* hybridization

A 424 bp fragment of the 16S rRNA gene of *Wolbachia* of *B. malayi* was amplified (forward primer 5′CAGCTCGTGTCGTGAGATGT, reverse primer 5′ CCCAGTCATGATCCCACTT) and cloned into a dual promoter PCRII plasmid (Invitrogen). After linearization of the plasmid, probes (anti-sense) and negative controls (sense) were prepared with Megascript T7 and Sp6 high yield transcription kits according to the manufacturer's suggested protocol (Ambion, Invitrogen). For labeling of the probe a biotin-16 dUTP mix (Roche, Indianapolis, IN, USA) was used during *in vitro* transcription. The plasmid template was then removed by DNase digestion (Roche). The probes were concentrated by ethanol precipitation, re-suspended in DEPC-treated water, and stored at −20°C until use.

For staining, 5 µm thin paraffin sections were deparaffinized and partially digested with pepsin HCl for approximately 7 minutes. Sections were hybridized at 60°C overnight in a humid chamber with 1 µg of rRNA probe in hybridization buffer (50% formamide, 5XSSC, 0.3 mg/ml yeast tRNA, 100 µg/ml heparin, 1× Denhart's Solution, 0.1% CHAPS and 5 mM EDTA). A stringency wash was performed at 60°C for 30 min, and detection was performed using the ‘*In situ* Hybridization Detection System’ (K0601, Dako) which uses alkaline phosphatase conjugated streptavidin to localize biotinylated rRNA probes. Sections were incubated for 20 min with streptavidin-AP conjugate at room temperature. BCIP/NBT substrate solution was added for 10 to 30 min to localize binding of the probes.

### DNA oligonucleotide probe fluorescence-based *in situ* hybridization (FISH)

Sections were deparaffinized and partially digested as described above and hybridized at 37°C overnight in a dark humid chamber using 200 ng of a custom made, labeled 30-mer antisense probe targeting the 16S rRNA of *Wolbachia* (wBm16S as, 5′Alexa 488-CAGTTTATCACTAGCAGT TTCCTTAAAGTC, Invitrogen). The complementary sense sequence was used as a negative control probe. One stringency wash was performed at 37°C for 30 minutes. Hybridization and stringency buffers were the same as described above. Finally sections were rinsed briefly in PBS and covered with a cover slip with ProLong Gold antifade reagent that contains DAPI (Invitrogen). This embedding reagent enables simultaneous fluorescence-based detection of condensed DNA in eukaryotic and prokaryotic organisms. Sections were examined using an Olympus-BX40 microscope equipped with the Olympus fluorescence filter 41001 (excitation 460–500 nm, emission 510–550 nm) for Alexa fluor or UN31000V2 (excitation 325–375 nm, emission 435–485 nm) for DAPI.

### Transmission electron microscopy

For ultrastructural analysis fixed samples were washed in phosphate buffer, embedded in agarose, and postfixed in 1% osmium tetroxide (Polysciences Inc.) for 1 hr as described previously [12]. Samples were then rinsed extensively in dH_2_0 prior to en bloc staining with 1% aqueous uranyl acetate (Ted Pella Inc., Redding, CA, USA) for 1 hr. Following several rinses in dH_2_0, samples were dehydrated in a graded series of ethanol and embedded in Eponate 12 resin (Ted Pella Inc.). Sections of 95 nm were cut with a Leica Ultracut UCT ultramicrotome (Leica Microsystems Inc., Bannockburn, IL,USA), stained with uranyl acetate and lead citrate, and viewed on a JEOL 1200 EX transmission electron microscope (JEOL USA Inc., Peabody, MA, USA).

## Results

### Localization of *Wolbachia* in larval *B. malayi*

**Figure 1:**
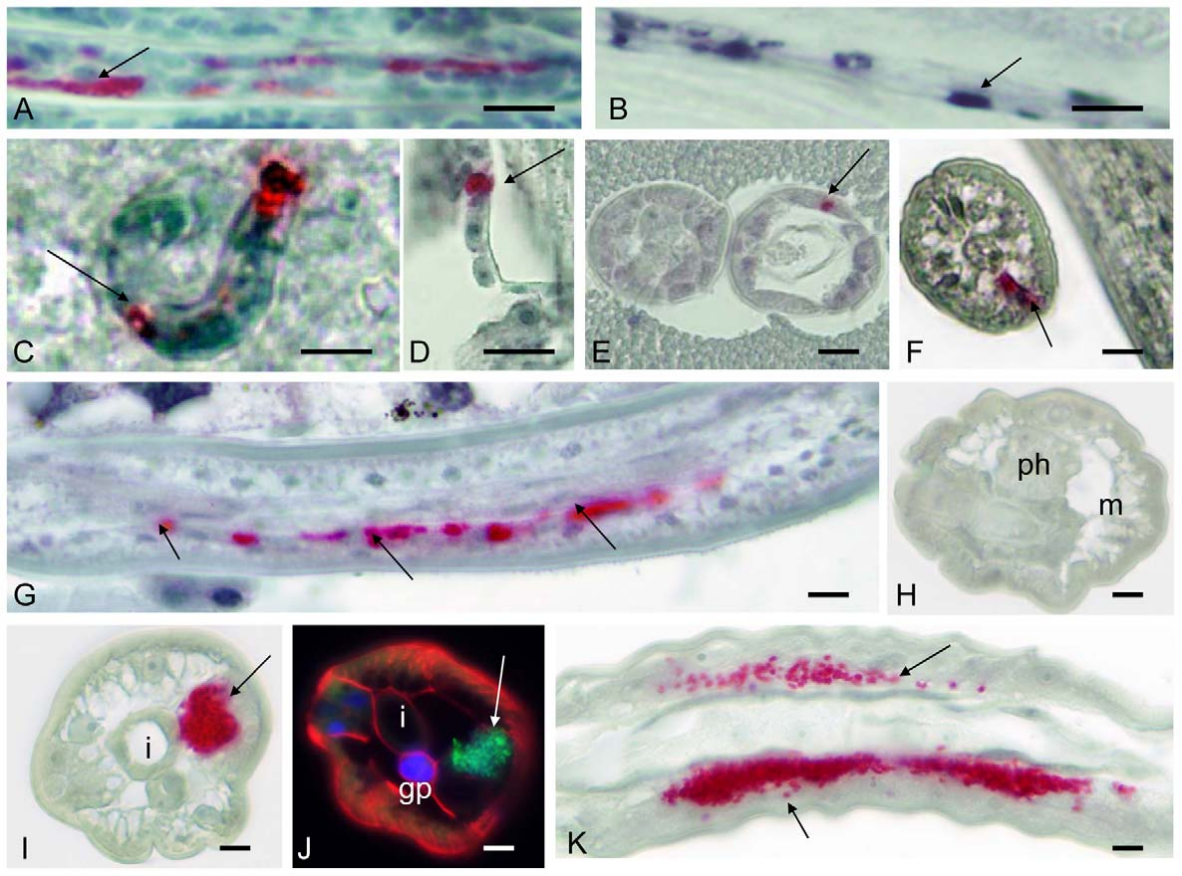
Detection of *Wolbachia* in larval *B. malayi* by immunohistology or by *in situ* hybridization. **A** Clusters of *Wolbachia* (arrow) in single cells of a stretched intrauterine microfilaria labeled by immunohistology using mab *Bm* WSP. **B** Consecutive section to **A**, but *Wolbachia* (arrow) were detected by *in situ* hybridization. **C** *Wolbachia* (arrow) in single cells of a microfilaria within the midgut of *A. aegypti* 2 h after the blood meal. **D** Clustered *Wolbachia* in a cell of a fragment of an L2 larva in the thorax of *A. aegypti* 3 d.p.i. **E** Cross-section of L2 larvae in the thorax of *A. aegypti* showing a cluster of *Wolbachia* (arrow) in the hypodermis (7 d.p.i.) **F** Cross section of isolated infective L3 larvae showing only a single *Wolbachia* cluster (arrow) in the cells of lateral chord (14 d.p.i). **G** Several clusters of *Wolbachia* (arrows) in the lateral chord in a longitudinal section of an infective L3 larva migrating through the abdomen of *A. aegypti* 14 d.p.i. **H** Cross-section of the anterior end of a 4^th^ stage larva (14 d.p.i. of a jird). No *Wolbachia* were detected. **I** Cross-section of the midbody region of a 4^th^ stage larva showing many *Wolbachia* (arrow) in one lateral chord. **J** Cross-section of the midbody region of another 4^th^ stage larva (wide field fluorescence microscopy) showing highly condensed DNA (blue DAPI stain) in the genital primordium and numerous *Wolbachia* (arrow) in the lateral chord similar to I. **K** Longitudinal section of the midbody region of a 4^th^ stage larva with many *Wolbachia* in the lateral chords (arrows). Cells in the pseudocoelomic cavity were free of *Wolbachia*. Ph, pharynx; gp, genital primordium; I, intestine; m, muscle. Scale bar 10 µm.

Different developmental stages were stained with mab *Bm* WSP (Fig. 1A, C–K). Results were confirmed by *in situ* hybridization or DAPI chromatin staining. Clusters of *Wolbachia* were detected in relatively few cells in microfilariae (Fig. 1A). The same staining pattern was observed by rRNA *in situ* hybridization with a probe for *Wolbachia* 16S rRNA (Fig. 1B). *Wolbachia* were sometimes detected in single cells of microfilariae within the midgut of mosquito vectors (Fig. 1C) or in sausage stage larvae and 2^nd^ stage larvae in the mosquito thorax (Fig. 1D, E), but most of the cells in these larval stages were free of the endobacteria. Even in infective 3^rd^ stage larvae the vast majority of cells were devoid of *Wolbachia* (Fig. 1F); *Wolbachia* in L3 were mainly present in the cells of the lateral chord, but not in internal organs (Fig. 1G).

The *Wolbachia* density at the anterior end of 4^th^ stage larvae was low at 2 weeks p.i. in the vertebrate host (3–5 days after the molt), and no endobacteria were detected in the tissue around the pharynx (Fig. 1H). In contrast, large numbers of *Wolbachia* were detected in the developing lateral chords of the L4 midbody region (Fig. 1I–K).

### Detection of *Wolbachia* during the development of adult female *B. malayi*

**Figure 2:**
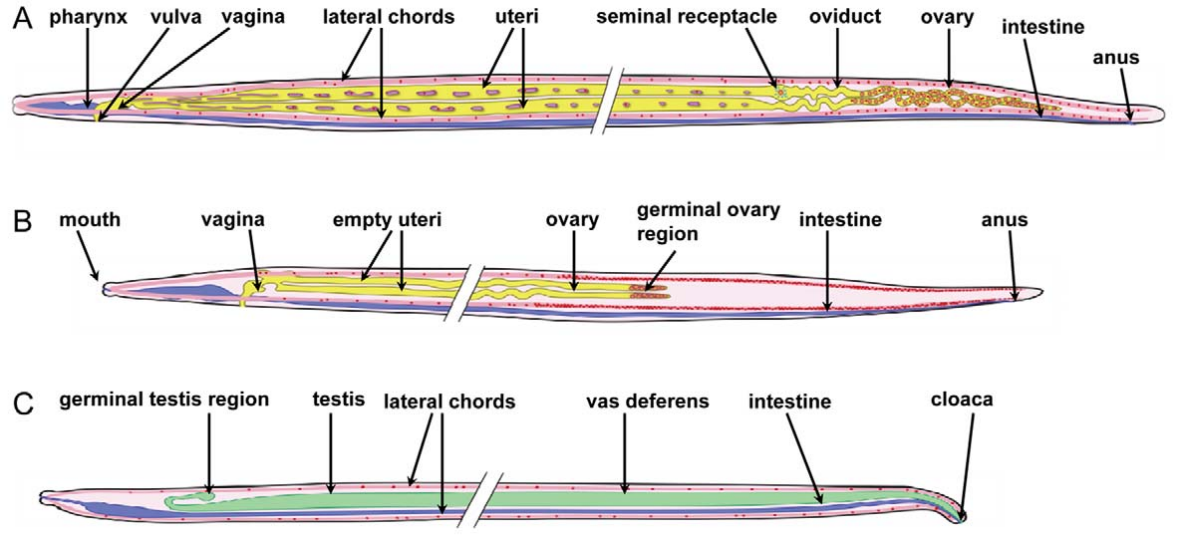
Schematic drawing the anatomy of adult stage *B. malayi* and distribution of *Wolbachia*. Hypodermis, muscles, median chords as well as nerve and secretory-excretory system are not shown. Proportions are estimates. Tissues and organ systems are simplified and the midbody region is interrupted for clarity. The lateral chords are shown dorsally and ventrally instead of laterally. **A** Adult microfilaria producing female (12 weeks p.i.). The body length is about 4 cm. *Wolbachia* (red dots) are localized mainly in the lateral chords, ovary and developing embryos. The lateral chords in the head (up to the vulva) of the worm rarely contain *Wolbachia*. **B** Adult stage, immature female (5 weeks p.i.) a few days after the 4^th^ molt. The body length is approximately 1.8 cm. *Wolbachia* are mainly localized in the lateral chords. Occasionally *Wolbachia* are attached to the ovaries or single endobacteria can be found within the ovary. **C** Adult spermatozoa producing male worm (12 weeks p.i.) with a total body length of about 2.5 cm. Spiculae are not shown. *Wolbachia* are localized in the lateral chords; *Wolbachia* remnants can be detected in parts of the *vas deferens* but not within spermatids or spermatozoa.

**Figure 3:**
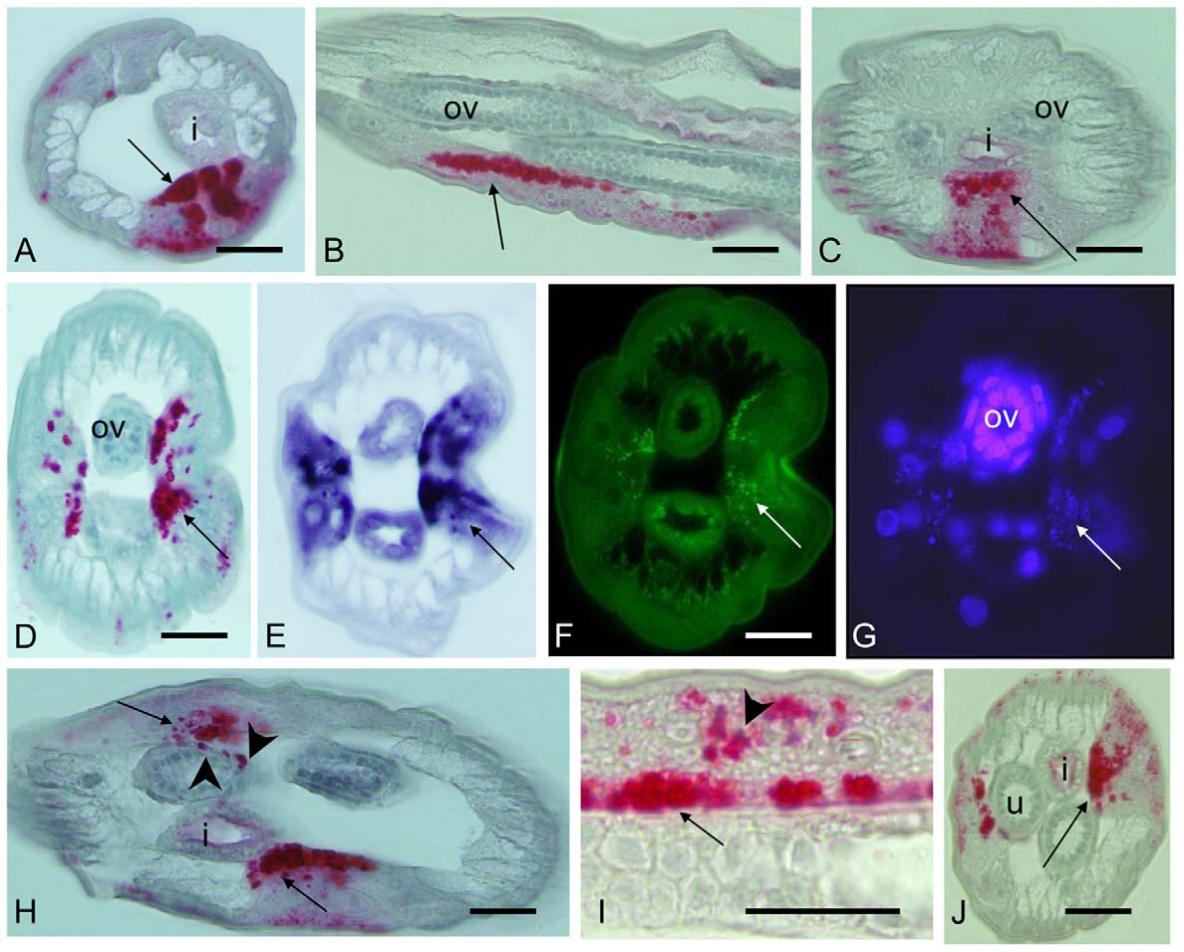
Detection of *Wolbachia* in immature female *B. malayi* at 5 weeks p.i. **A**–**D**, **H**–**J** immunohistology using mab *Bm* WSP. **A** Cross-section in the posterior part of the female showing massive accumulation of *Wolbachia* (arrow) in the lateral chord. **B** Longitudinal section of the distal tip showing *Wolbachia*-free growing ovaries and numerous clusters of *Wolbachia* (arrow) in the lateral chord. **C** Cross-section showing asymmetric distribution of large amounts of *Wolbachia* (arrow) in the lateral chord and *Wolbachia*-free ovaries. **D** Large numbers of *Wolbachia* (arrow) in close proximity to one ovary branch. Ovaries are still not infected. **E**–**G** Consecutive sections to **D**. **E** 16S rRNA *in situ* hybridization. **F** 16S oligonucleotide FISH. **G** DAPI stain showing highly condensed chromatin in the ovary and in *Wolbachia* in the lateral chord (arrow). **H** Cross-section showing not only *Wolbachia* clusters in the lateral chords (arrow) but also at the edge of one ovary (arrow head). **I** Sagital section through lateral chord and ovary showing large numbers of *Wolbachia* clusters (arrows) in the lateral chords aligned at the ovary and some *Wolbachia* clusters in the middle of the chord (arrow head). **J** Cross-section of the midbody region showing large numbers of *Wolbachia* in the lateral chord (arrow) and empty uterus branches. i, intestine; u, uterus; ov, ovary. Scale bar 25 µm.

In order to understand the distribution of *Wolbachia* in adult worms it is crucial to recall the anatomy and development of reproductive organs of filarial worms [13], [14]. The genital opening (vulva) lies close to the anterior end of the female worm, approximately at the level of the esophagus (Fig. 2A, B). The vagina leads into the bifurcated uterus which ends in the seminal receptacles. Theses organs are linked by oviducts with two ovaries that have an anterior growth zone, a maturation zone in the middle, and a posterior germinative zone. At 5 weeks p.i. in the vertebrate host, young adult female *B. malayi* worms are approximately 1.8 cm long and still growing. At that time point a massive accumulation of *Wolbachia* was observed, mainly in the lateral chords. Increased numbers of *Wolbachia* were observed in the lateral chords in the posterior end of the female which was still free of ovaries (Fig. 3A). Sections of the posterior part of the ovaries showed large numbers of *Wolbachia* in the adjacent lateral chords, but the ovaries themselves were free of *Wolbachia* (Fig. 3B, C, 4A). In the oocyte maturation and growth zones of the ovaries, *Wolbachia* were oriented within the lateral chords towards the pseudocoelomic cavity (Fig. 3D–G), and some sections showed *Wolbachia* in the periphery of the ovary (Fig. 3H, 4B, D, F). Two distribution patterns of *Wolbachia* were found in the lateral chords. Scattered *Wolbachia* were present in the apical part of the chords, and numerous clusters of *Wolbachia* were present basal border of the hypodermal chords adjacent to the ovaries (Fig. 3I). A similar staining pattern for *Wolbachia* was observed in the lateral chords in the midbody region of 5 week old females, but their empty uterus branches were always free of *Wolbachia* (Fig. 3J). In 8 week old female worms, less *Wolbachia* were detected in the lateral chords, but the mature ovaries in the posterior part of the worms were heavily infected with *Wolbachia* (Fig. 5A). These worms contained developing microfilariae, and the ovaries showed strong staining of nuclear chromatin (as determined by DAPI). Morula stage embryos were observed in the uterus with many *Wolbachia*, while in this region the number of endobacteria in the lateral chords was lower than in the distal parts of the lateral chords.

**Figure 4:**
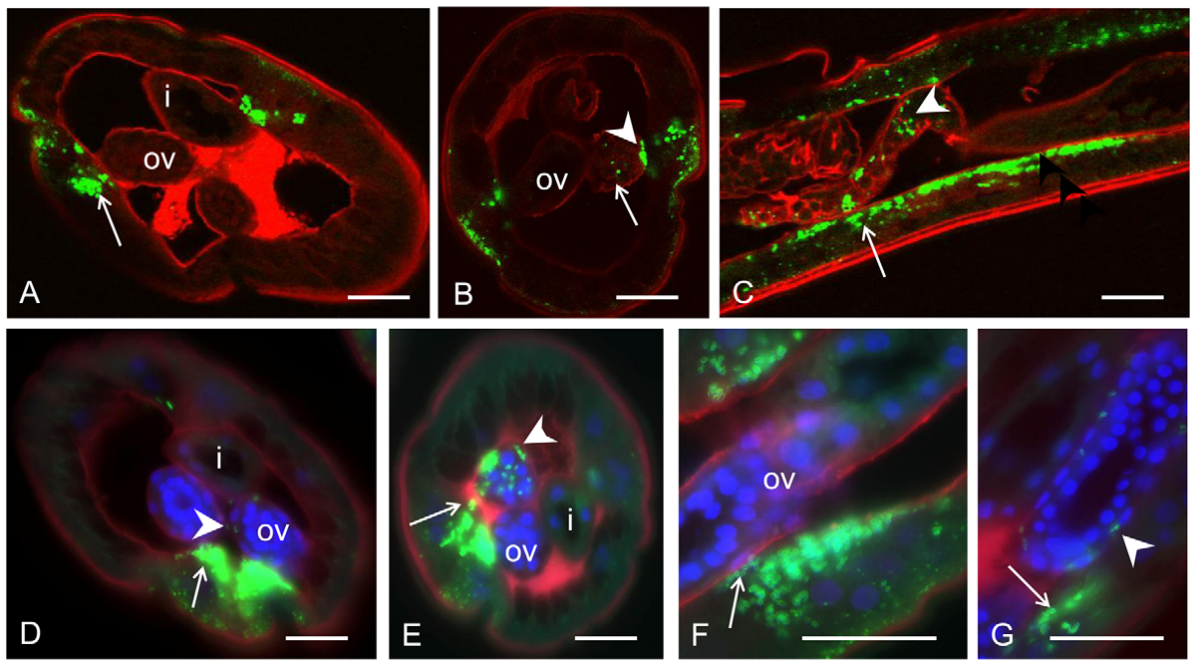
Detection of *Wolbachia* in female *B. malayi* at 5 weeks p.i. by advanced microscopy techniques. Confocal laser scanning microcopy (LSM) and wide field fluorescence microscopy (WFFM) were used together with mab *Bm* WSP (green) to label *Wolbachia* and a WGA 633 stain (red) to visualize membranes. WFFM contained also a filter for DAPI to detected concentrated DNA. **A** Cross-section showing large amounts of *Wolbachia* in the lateral chords close to the ovaries (arrow). Some *Wolbachia* were also detected in the hypodermis. For full scans of LSM images and 360° rotation see suppl. material (LSM). **B** Another cross-section showing a more advanced stage of infection with endobacteria attached to the ovary membrane (arrow head) and already in the ovaries (arrow) (LSM). **C** Longitudinal section showing *Wolbachia* lining up in the lateral chords in the vicinity of the ovary (arrow) or within one ovary branch (arrow head)(LSM). **D** WFFM showing *Wolbachi*a approaching ovaries with highly condensed chromatin (blue DAPI stain). **E** Another cross-section showing *Wolbachia* (arrows) attached to the ovaries (blue DAPI stain). One ovary branch is already *Wolbachia* (arrow head) infected while the other is still free (WFFM). **F** Close-up of a longitunial section showing *Wolbachia* (arrow) directly at the ovary membrane (red) (WFFM). **G** Another close-up showing *Wolbachia* in the lateral chords close to the ovary (arrow) or already in the somatic cells of the ovary (arrow head) (WFFM). Staining for WSP provides a characteristic donut shaped pattern of a *Wolbachia* surface protein. Ov, ovary; i, intestine. Scale bar 30 µm.

**Figure 5:**
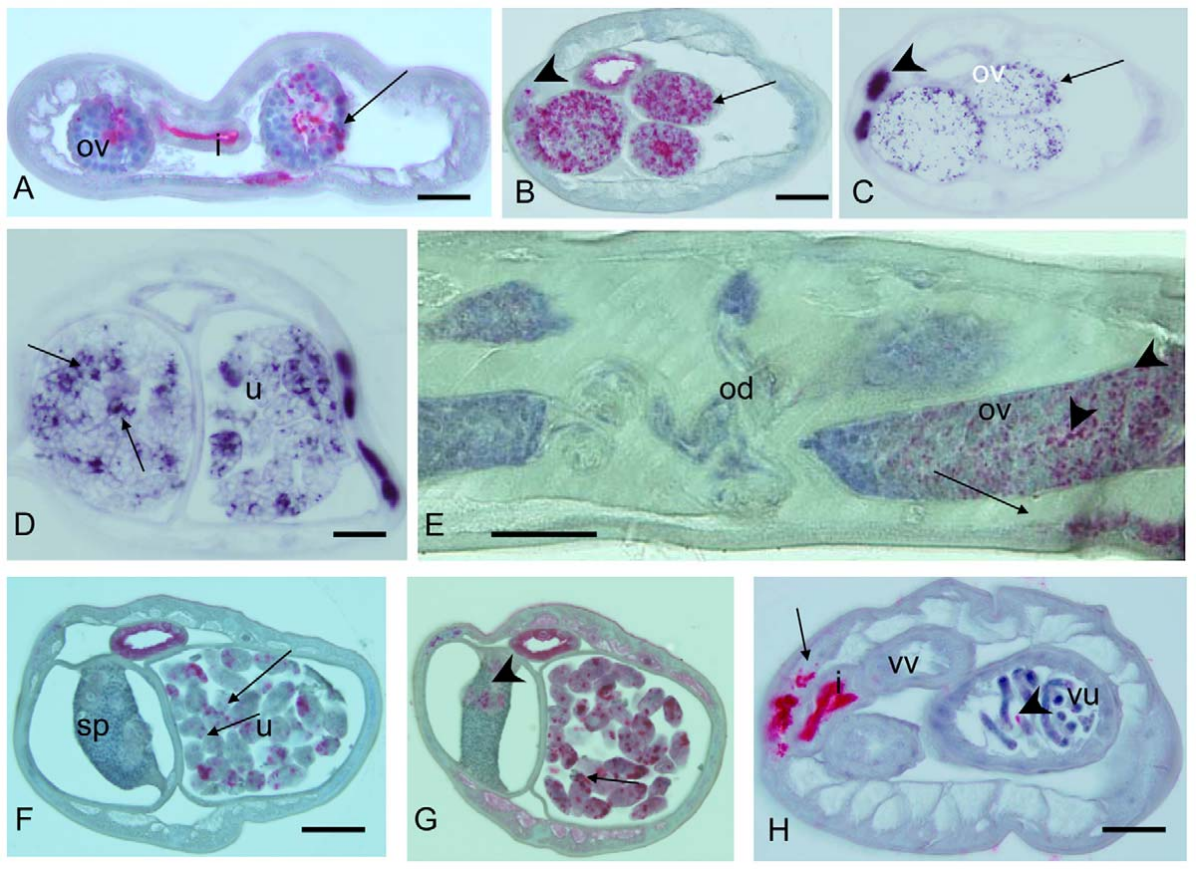
Detection of *Wolbachia* in adult female *B. malayi* at 8 and 12 weeks p.i. In A, B, E, F and H *Wolbachia* were labeled by mab *Bm* WSP. **A** Cross-section of a distal end of a worm 8 weeks p.i. showing *Wolbachia* infected ovaries and some *Wolbachia* clusters in the lateral chord. **B**–**H** *B. malayi* 12 weeks p.i. **B** Several cross-sections of the ovaries showing massive *Wolbachia* staining (arrow), but only weak labeling in the adjacent lateral chord (arrow head). **C** Consecutive section to **B**, but *in situ* 16S rRNA detection confirmed the large number of *Wolbachia* in the ovaries with much stronger labeling in the adjacent lateral chord (arrow head). **D** Cross-section of the midbody region shows both uterus branches full of morula stage embryos and *Wolbachia* (arrow, 16S rRNA). **E** Longitudinal section in the region of the transition of the ovaries to the tubular oviduct. Large numbers of *Wolbachia* are visible in the growth zone of the ovaries (arrow heads) and the adjacent lateral chords (arrow) but not in the oviduct. **F** Cross-section showing one uterus branch with *Wolbachia* positive (arrow) morula stage embryos and numerous spermatozoa in the seminal receptacle surrounding a few egg cells. Thin lateral chords show only scattered *Wolbachia*. **G** Consecutive section to **F** stained with polyclonal *Brugia pahangi* WSP antisera [39] shows *Wolbachia* in the egg cells (arrowhead). **H** Cross-section of the region close to the female genital opening. Some *Wolbachia* are visible in the lateral chord (arrow) and in the stretched microfilariae (arrowhead) in the vagina uterine, but the muscular vagina vera is free of microfilariae and *Wolbachia*. Ov, ovary; u, uterus; od, oviduct; sp, spermatozoa; vv, vagina vera; vu, vagina uterina. Scale bar 25 µm.

In 12 week old females numerous *Wolbachia* were observed in the lateral chords and the posterior parts of the ovaries, but bacteria densities in these areas were lower than in the lateral chords adjacent to the anterior ovary and oviduct (Fig. 5B,C,E, H). Numerous *Wolbachia* were detected in morula stage embryos in the uterus, but only a few were detected in the lateral chords of females at that level (Fig. 5D, F, G). Intrauterine spermatozoa surrounding degenerated oocytes in the seminal receptacle were free of *Wolbachia*, but serial sections showed some *Wolbachia* in the oocytes in this area (Fig. 5F,G). Stretched microfilariae in the vagina uterina contained *Wolbachia* in some cells, but the numbers were low compared to those in morula stage embryos. This suggests that *Wolbachia* may be necessary for rapid cell division which occurs in developing embryos but not in stretched microfilariae. The distribution of *Wolbachia* in the lateral chords was often asymmetrical, and this depended on the proximity to the reproductive system, body region and on the age of the worm.

### Detection of *Wolbachia* during the development of adult male *B. malayi*

**Figure 6:**
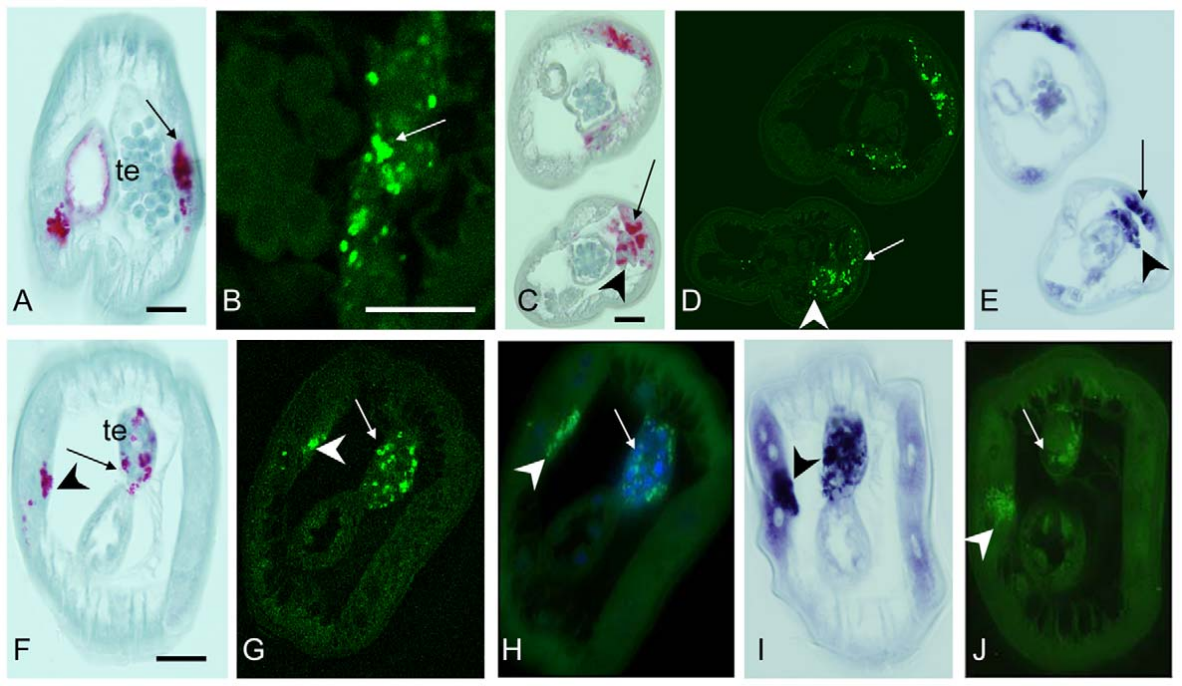
Detection of *Wolbachia* in immature male *B. malayi* at 5 weeks p.i. In **A** and **C** *Wolbachia* were labeled by mab *Bm* WSP). **A** This cross-section of the midbody region shows primary spermatogonia in the testis and numerous *Wolbachia* (arrow) in the lateral chords. **B** LSM of a consecutive section of A. Individual clusters of *Wolbachia* (arrow) can be identified. **C** Two cross-sections more distal compared to **A** with *Wolbachia* in the lateral chords (arrow) and in tissue attached to the testis (arrow head). **D** Consecutive section to **C** examined by LSM. **E** Consecutive section to **C** stained by *in situ* rRNA hybridization confirming the staining pattern. **F**–**J** Serial cross-sections showing many *Wolbachia* in the lateral chords (arrowheads) and in the spermatogonia (arrows). **F** mab *Bm* WSP. **G** Also mab *Bm* WSP but LSM **H** Same staining as **G** but WFFM with DAPI stain of testis. **I** 16S *in situ* rRNA hybridization. **J** 16S oligonucleotide FISH. te, testis. Scale bar 25 µm.

**Figure 7:**
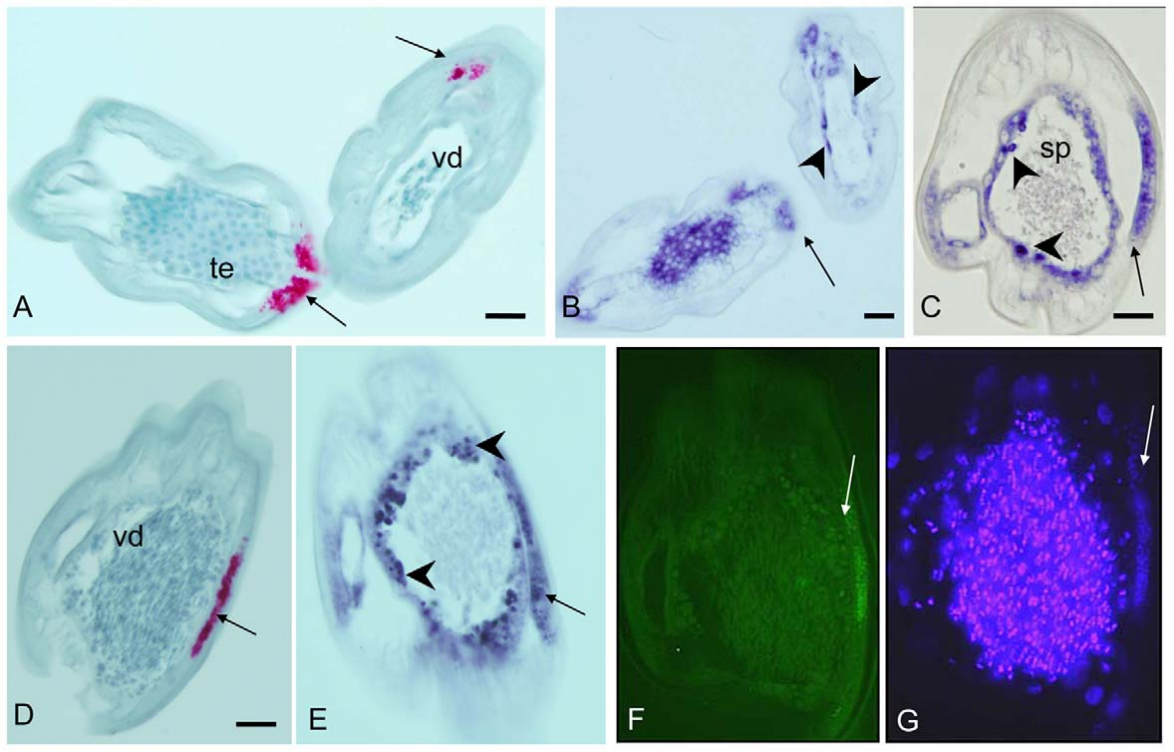
Detection of *Wolbachia* in male *B. malayi* at 12 weeks p.i. In **A** and **D** *Wolbachia* were labeled by mab *Bm* WSP). **A** Two cross-sections demonstrating *Wolbachia* in the lateral chords (arrows). One section is in the midbody region showing developed spermatogonia and numerous *Wolbachia* in the lateral chord, while the other section is in the more muscular distal end of the worm showing spherical spermatids in the *vas deferens* and fewer *Wolbachia* in the lateral chord. **B** Consecutive section to **A**, but stained with 16S rRNA in situ hybridization. In contrast to **A** some staining for *Wolbachia* rRNA is also detected in the testis (arrowheads) and at the border of the *vas deferens* (arrow heads). **C** Cross-section of the distal part of another male worm showing *Wolbachia* 16S rRNA labeling at the epithelium of the *vas deferens* (arrow heads) and in the lateral chord (arrow). Spermatozoa were never labeled. **D**–**G** Consecutive cross-section through the terminal end showing the *vas deferens* containing fully developed spermatids in transition to spermatozoa. *Wolbachia* are detected in the lateral chord (arrow), but not in the spermatids. **D** mab *Bm* WSP. **E** 16S rRNA *in situ* hybridization. Similarly to **B**, staining is also observed at the border of the *vas deferens* (arrowheads). **F** 16S oligonucleotide FISH. Although *Wolbachia* can be easily identified in the lateral chord, granular staining at the membrane of the *vas deferens* is difficult to recognize. **G** DAPI stain, again *Wolbachia* can be easily identified in the lateral chord, but granular staining at the membrane of the *vas deferens* is hard to differentiate. Sp, spermatozoa; te, testis; vd, vas deferens. Scale bar 25 µm.

The genital opening of the male worm lies at the posterior end and forms with the anus a cloaca (Fig. 2C). This is in stark contrast to the anatomy of females. A single *vas deferens* leads into a seminal vesicle that is connected to the testis; this can be subdivided into a growth zone, a maturation zone, and a germinative zone. In parallel to the distribution of *Wolbachia* in females, large numbers of endobacteria were observed in the lateral chords of 5 week old males, while the growing sections of the testes in the midbody region were free of *Wolbachia* (Fig. 6A). However, *Wolbachia* were present in 5 week males near the testes (Fig. 6B–E) and in the middle part of the testis itself (Fig. 6 F–J). No *Wolbachia* were detected within the *vas deferens* by immunohistology (Fig. 7A, D). In contrast, *Wolbachia* 16S rRNA was detected by *in situ* hybridization in the testis tissue surrounding the spermatocytes and in the periphery of the *vas deferens* that contained spermatids (Fig. 7B, C, E). *Wolbachia* were never observed in the spermatids or the spermatozoa.

### Comparison of morphological detection methods

Comparison of four different methods on consecutive sections (Figs. 3 D–G; 6 C–E; 6 F–J; 7A,B; 7D–G) revealed almost identical staining patterns for *Wolbachia* by immunohistology with mab *Bm* WSP, by i*n situ* hybridization (using FISH and RNA *in situ* to detect *Bm Wolbachia* 16S rRNA), and by DAPI staining. Differences between immunohistology and 16S rRNA *in situ* detection were occasionally observed (Figs. 5B, C; 7A, B; 7D, E). In these cases *in situ* hybridization detected a strong *Wolbachia* 16S rRNA signal, while no or very little *Wolbachia* surface protein was detectable by immunohistology. This may indicate a small difference of gene expression pattern or of gene product stability of both markers, but was not noticed as confounding factor. In addition, the intestine of *B. malayi* was sometimes nonspecifically labeled by immunohistology because of endogenous alkaline phosphatase (e.g. Fig. 5A, F–H). This did not occur with *in situ* staining.

The mab *Bm* WSP immunohistology assay detects a protein on the surface of *Wolbachia*, and it is possible that this protein is not present on all *Wolbachia* cells. In contrast, the *in situ* hybridization assay detects expression of 16S rRNA in the cytoplasm of *Wolbachia*. Small subunit rRNA is known to be highly expressed during the exponential growth phase of bacteria, and that has been used as marker for viability [15]. Therefore the *in situ* assay is an excellent marker for *Wolbachia* growth, and it may be suitable for assessing both the presence and viability of *Wolbachia*. DAPI staining, which detects A-T rich regions in DNA, is an easy and quick method to detect *Wolbachia* in the lateral chords, since this syncytial tissue usually does not contain condensed filarial chromosomes (Figs. 3G; 7G). However, it is difficult to identify *Wolbachia* by DAPI staining in areas with condensed filarial chromosomes such as ovaries or in spermatids within the *vas deferens* (Figs. 3G; 7G). This problem can be solved by combining the DAPI stain for condensed DNA with immunohistology (Figs. 2J, 4D–G; 6H). This permits visualization of *Wolbachia* in the vicinity of filarial nuclei.

### Confocal laser scanning microscopy

Confocal laser scanning microscopy was used to study the three dimensional distribution of *Wolbachia* in larvae and in developing reproductive tissue of young adult worms. Although *Wolbachia* numbers were increasing in the lateral chords in 4^th^ stage larvae, no *Wolbachia* were observed in developing reproductive organs in L4. The higher resolution of LSM confirmed heavy *Wolbachia* loads in the lateral chords of young female worms (5 weeks) and relatively few endobacteria in the hypodermis (Fig. 4A). Entire oocytes could be examined for *Wolbachia*, because the size of oocytes is less than 5 µm and the scanned slices were 0.8 µm thick which is about the size of an endobacteria. The confocal examination of the distal end of the ovaries in 5 week old females confirmed the absence of *Wolbachia* from primary oocytes (Fig. 4A, D). A full LSM scan and rotation of the section show that *Wolbachia* were present also in the hypodermal pouches that form longitudinal lines in 5 week old female worms (video S1). A membrane stain helped to demonstrate that some *Wolbachia* were attached to the external membrane around the proximal ovary while other bacteria were actually in the ovary (Fig. 4B, C, videos S2, S3). The latter *Wolbachia* were always in the vicinity of large clusters of *Wolbachia* in the lateral chords adjacent to the ovaries in developing adult female worms (Fig. 4B, C). Wide field fluorescence microscopy using FITC labeled mab *Bm* WSP with a membrane stain and an overlay of the DAPI nuclear stain showed that *Wolbachia* are attached to the ovary membranes (Fig. 4D, E, F, G). It is possible that these endobacteria invade the ovaries of young females from the lateral chords. *Wolbachia* distribution in the developing ovaries was not uniform; in some cases, one branch was infected while the other branch was *Wolbachia* free (Fig. 4E).

### Ultrastructural studies of *Wolbachia* in developing reproductive tissue

**Figure 8:**
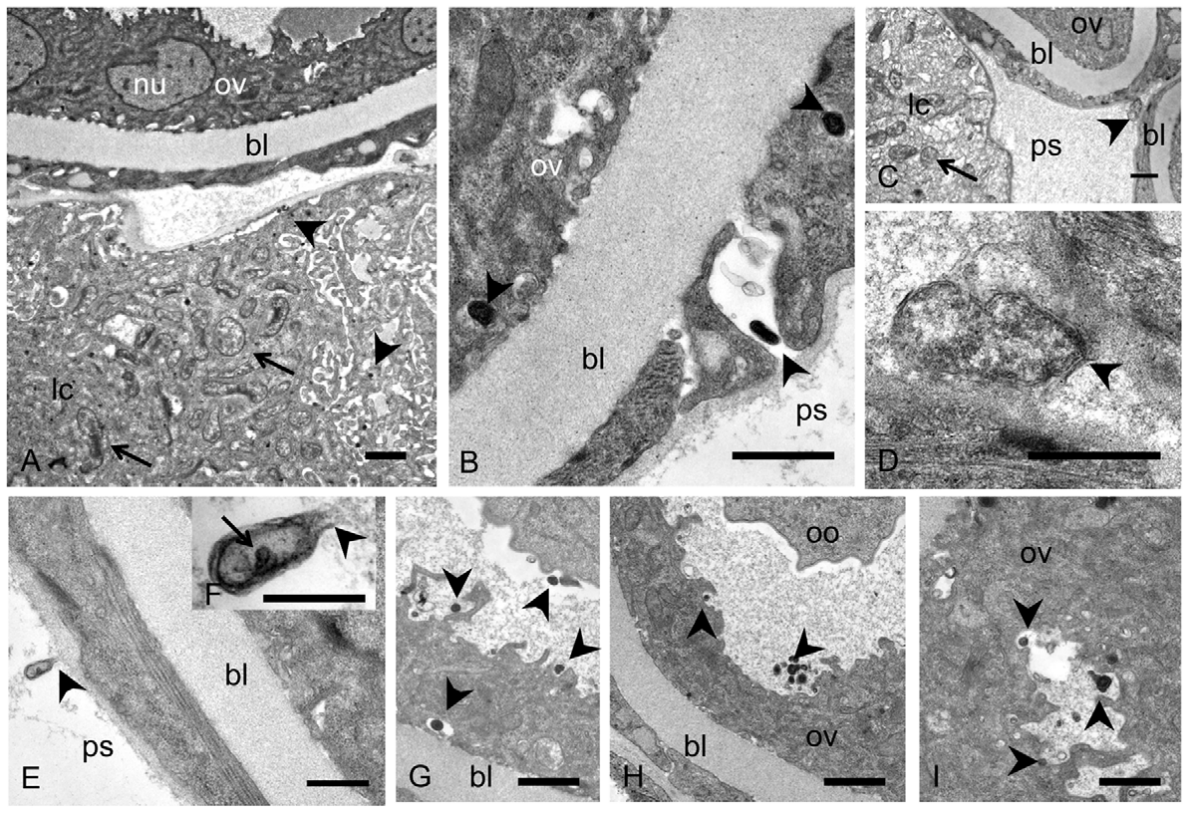
Transmission electron microscopy of young adult female *B. malayi* 5 weeks p.i. **A** Numerous *Wolbachia* (arrow) in the lateral chord in the vicinity of developing ovaries. A number of small, electron dense *Wolbachia* can be detected in more loosened tissue (arrow heads). **B** Vacuolized epithelium surrounding the ovary with electron dense *Wolbachia* (arrow heads) on both sides of the basal lamina. **C** Numerous *Wolbachia* in the lateral chord (arrow) and a bacilli-shaped, extracellular *Wolbachia* in the pseudocoelomic cavity (arrow head). **D** Magnification of **C**, showing the *Wolbachia* cell membrane (arrow head). **E** *Wolbachia* (arrow head) docking to the outer ovary epithelium. **F** Magnification of the endobacterium in **E** showing loosened cell membrane at the apical end (arrow head) and a dense central inclusion (arrow). **G** Small electron dense *Wolbachia* (arrow heads) of the oogonia in the proximal part of the ovary. **H** Single and clusters of electron dense, extracellular *Wolbachia* (arrow heads) in the ovary. **I** Electron dense *Wolbachia* (arrow heads) in vacuolized ovary tissue. Bl, basal lamina; lc, lateral chord nu, nucleus; ov, ovary; ps, pseudocoelomic cavity. Scale bar 0.5 µm.

Studies of the midbody region of 5 week old worms by transmission electron microscopy confirmed the presence of *Wolbachia* in the vicinity of developing reproductive tissues. Numerous rod-shaped and spherical *Wolbachia* were detected in the lateral chords in females, especially in adult worm tissues that are adjacent to developing ovaries. In some areas the hypodermal chord tissue was loose and vacuolized (Fig. 8A). The epithelial cells surrounding the basal lamina of the ovaries were occasionally also strongly vacuolized indicating tissue degeneration, and small, electron dense *Wolbachia* were detected in these vacuoles (Fig. 8B). Occasionally extracellular *Wolbachia* were seen in the pseudocoelomic cavity docking to the edge of the ovaries (Fig. 8C, D) or attached to the outer ovarian tissue (Fig. 8E, F). While most of the *Wolbachia* in the lateral chords were rod-shaped or spherical and up to 1 µm in length and 0.5 µm in diameter, the endobacteria in the pseudocoelomic cavity were condensed, bacillary in shape and only 0.15 to 0.5 µm in length (Fig. 8G–I). Within the ovaries, these small *Wolbachia* forms were observed in large vacuoles or in loose ovarian tissue (Fig. 8G, I) either as single bacteria or in groups (Fig. 8H).

**Figure 9:**
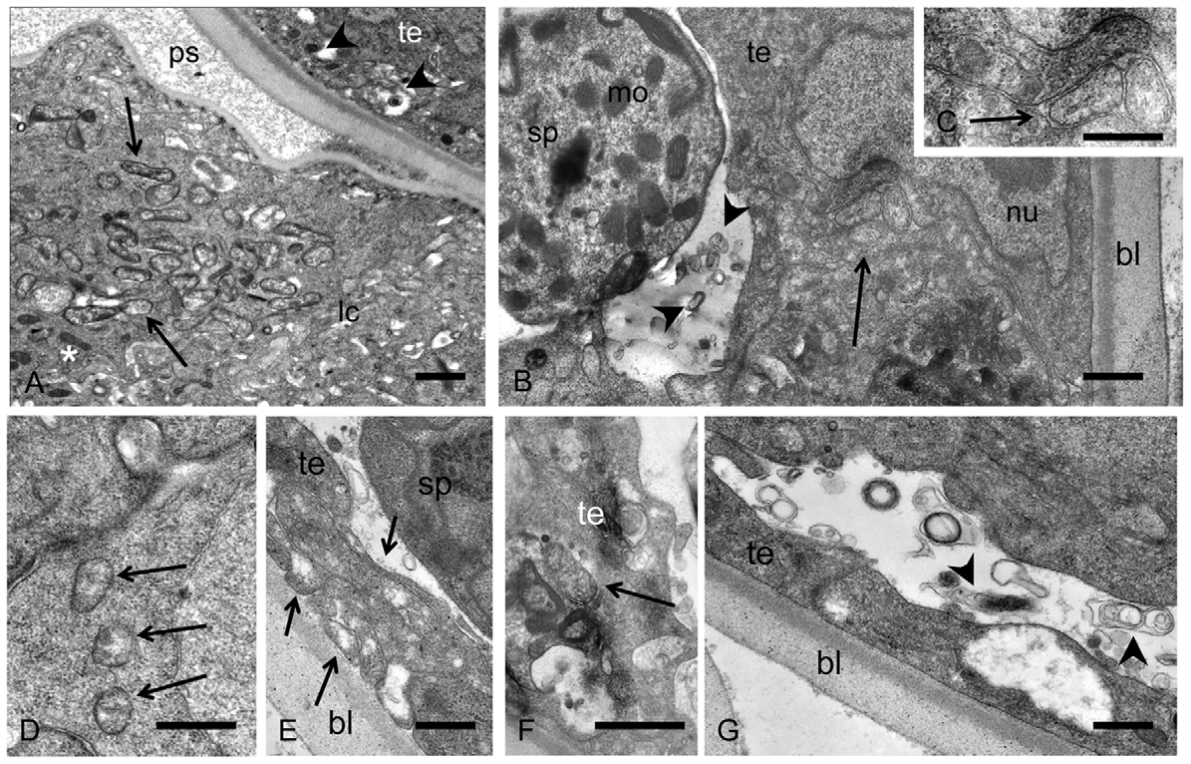
Transmission electron microscopy of young adult male *B. malayi* 5 weeks p.i. **A** Numerous *Wolbachia* (arrows) are observed in the lateral chords close to the testis. Single dark *Wolbachia* (arrow head) are found in the testis close to the membrane. Mitochondria (asterisk) are found in the periphery of the lateral chord. **B** *Wolbachia* (arrow) in the inner testis epithelium in the vicinity of a spermatid. Large amounts of membranous material (arrow heads) can be observed in the testis lumen in the vicinity of the testis epithelium. **C** Magnification of B showing intracellular *Wolbachia* (arrow). **D** Another sample showing *Wolbachia* (arrows) in the inner testis epithelium. **E**–**G** Pleomorphic *Wolbachia* (arrows) in vacuolized testis tissue. Membranous material (arrow head) can be seen in extracellular spaces. Ps, pseudocoelomic cavity; lc, lateral chord; sp, spermatide; mo, membranous organelle; nu, nucleus; bl, basal, lamina; te, testis. Scale bar 0.5 µm.

In 5 week old male worms large clusters of large, rod-shaped or spherical *Wolbachia* were observed in the lateral chords in the vicinity of the testis (Fig. 9A). Small, bacillary *Wolbachia* forms were sometimes observed in the testis tissue. At the caudal end of the testis, close to the transition to the *vas deferens*, *Wolbachia* were observed in the inner tissue, sometimes in the vicinity of peripheral spermatids (Fig. 9B,C, D). These spermatids can be easily identified and differentiated from mature spermatozoa by their compact membranous organelles and the absence of major sperm protein complexes. Large amounts of membranous material were observed in the lumen between the spermatids and the inner testis epithelium. This material resembles degenerating *Wolbachia* (Fig. 9B, E–G) as they have been described previously [16]. *Wolbachia* were unambiguously identified in the reproductive tissue of young male worms, but not in the spermatids or spermatozoa.

## Discussion

Immunohistology has been extensively used to study *Wolbachia* and their clearance following chemotherapy in *O. volvulus*. Compared to *O. volvulus*, mature *B. malayi* have a thinner hypodermis and less pronounced lateral chords, and this can make the detection of *Wolbachia* more difficult. Our results demonstrate that the distribution and density of *Wolbachia* vary in different tissues and developmental stages. Our results are consistent with those from a PCR study that reported low amounts of *Wolbachia* DNA in vector stages and larger amounts in mammalian stages [4]. McGarry and co-workers reported an exponential increase in *Wolbachia* DNA in transmitted *B. malayi* L3 larvae as early as 7 days p.i. We detected large amounts of endobacteria by histology in the lateral chords of the midbody region in L4 larvae (14 d.p.i.). More *Wolbachia* were present in young adult worms at 35 d.p.i. in most parts of the lateral chords and also in an uneven distribution in the hypodermis.

Observations on *Wolbachia* density and tissue localization may lead to hypotheses regarding their potential function in filarial worms. Antibiotic treatment experiments have suggested that *Wolbachia* may play a crucial role in the molting process of filarial parasites [17], [18], [19], [20]. It appears clear that if *Wolbachia* have a direct function during molting, this function does not require localization in the vicinity of the filarial cuticle, since our localization results show that *Wolbachia* are not located near the cuticle during or immediately after molting. The distinct age and tissue specific distribution patterns of *Wolbachia* suggest also that the bacteria are not likely to be needed for housekeeping functions in all cell types of filarial nematodes. The absence of *Wolbachia* in the filarial nervous system, muscles, or the digestive systems suggests that *Wolbachia* are not needed for these functions. In adult worms the majority of mitochondria can be found in the periphery of the lateral chords, while the majority of *Wolbachia* are localized in or near the reproductive system. The differential distribution of *Wolbachia* and mitochondria within the lateral chord of filarial parasites has been reported previously [21]. Especially to the female worms the localization of *Wolbachia* in the lateral chords in vicinity of the reproductive system implies an important role of endobacteria for embryogenesis and intrauterine development. In agreement with this hypothesis tetracycline treatment to deplete *Wolbachia* in developing filarial worms has been shown to affect mainly females and causes a male-biased sex-ratio [20], [22].

The *Wolbachia* genome in *B. malayi* encodes complete pathways for the biosynthesis of nucleotides, riboflavin, flavin adenine dinucleotide and heme, which are missing or incomplete in the filarial genome [23]. A high demand for gene products (which may not be taken up from the mammalian host) from these pathways might be especially necessary during the development of the reproductive system in young adult worms. Furthermore, the phylogenetically old and tight association of filarial nematodes with *Wolbachia* during reproduction may have led to additional interdependencies that account for their mutualistic relationship. As hypothesized for *Wolbachia* in insects, it is possible that *Wolbachia* in filarial nematodes are especially important for pre-meiotic mitosis, meiosis, and meiosis associated processes [24], [25], [26], [27].

A recent study examined the dynamics of *Wolbachia* during intrauterine embryogenesis of *B. malayi* using *Caenorhabditis elegans* embryogenesis as a framework for the analysis [5]. Asymmetric *Wolbachia* segregation was observed that could explain the concentration of *Wolbachia* in the hypodermal chords. The early differential distribution of *Wolbachia* within embryonic cells corresponds well with the strong tissue specific distribution in later development described in our study. However, the authors also hypothesized that the asymmetric segregation pattern may be responsible for the presence of *Wolbachia* in the female germline [5]. This is in contrast to our results which clearly demonstrate the absence of *Wolbachia* in male and female reproductive tissue from the third stage larvae to the young adult worms. Since it is difficult or impossible to identify the germline cells or gender of microfilariae, vector stage first stage larvae, and second stage larvae of *B. malayi*, we cannot be sure when during development *Wolbachia* are lost in these cells.

The terminal ends of *Brugia* ovaries form the germinative zones which contain the mitotic growing oogonia [28]. Our study showed that these areas were free of *Wolbachia* in growing, young adult worms. Our results suggest that *Wolbachia* from adjacent lateral chords may cross tissue zones to infect cells in maturation zone 1 (which mainly contains primary oocytes in the pachytene stage of meiotic prophase I) and in maturation zone 2 (which contains oocytes in the remaining phases of meiosis I). The germinative zones of the ovaries seem to be populated by *Wolbachia* over a period of approximately three weeks following the L4–L5 molt. Large numbers of *Wolbachi*a were present in the maturation zones of eight week or older female worms, while the attached growth zones which contain the secondary oocytes and the oviducts contained lower numbers of *Wolbachia* (see Fig. 5E). Fertilization precedes meiosis II in filarial nematodes [28]. *Wolbachia* were detected in secondary oocytes surrounded by spermatozoa and unfertilized oocytes within the seminal receptacle in mature females (see Fig. 5F, G).

The picture was similar in male worms. *Wolbachia* were not observed in the germinative zone of the testis. It is possible that *Wolbachia* from the lateral chords infect the primary spermatocytes in maturation zone 1, which are mostly in the pachytene stage of prophase of meiosis I [29]. This report is the first detection of *Wolbachia* in primary spermatocytes of developing male filarial nematodes. Although mature male worms have been previously examined for *Wolbachia*, prior studies did not report infection of the testis [30]. This is not contradictory to our findings, since *Wolbachia* appear to only infect the testis of immature adult stage *B. malayi* males and such worms were not studied previously. The spermatocytes of the adjacent growth zone and maturation zone 2 are difficult to differentiate morphologically, but larger secondary spermatocytes that have completed meiosis and the spherical spermatids which enter the *vas deferens* can be distinguished. *Wolbachia* were never seen in the spermatids or the mature spermatozoa. However, our *in situ* hybridization results clearly indicated the presence of *Wolbachia* 16S rRNA in the periphery of the seminal vesicle. This was confirmed by electron microscopy that showed *Wolbachia* in the inner epithelium of the testis or *vas deferens*, but not in the spermatids. These data may suggest that high *Wolbachia* densities are correlated with condensed chromatin and *Wolbachia* may be involved in chromosome segregation of filarial nematodes.

Our ultrastructural studies of young adult *B. malayi* confirm that *Wolbachia* are highly pleomorphic. This pleomorphism was recognized shortly after the discovery of endobacteria in filarial nematodes, and it has been suggested that *Wolbachia* may have a *Chlamydia*-like life cycle with small dense bodies as potential infectious forms [30], [31]. *Chlamydia* and filarial *Wolbachia* both have an obligatory intracellular life style and a small genome size due to the loss of a number of essential biosynthetic pathways. Both bacterial groups lack cell walls but retained a functional lipid II biosynthesis pathway [32]. It is also possible that *Wolbachia* share the requirement of *Chlamydia* for host cell sphingolipids supplied by the host cell Golgi apparatus and multivesicular bodies for activation [33]. Clearly, further studies are needed to assign functions to different morphological forms of *Wolbachia* during the filarial life cycle.

Based on our results we hypothesize that the genital primordium in larval *B. malayi* is devoid of *Wolbachia* and that reproductive tissues in young adult worms become infected with *Wolbachia* from adjacent lateral chords which have many *Wolbachia*. Prior studies have shown that newly introduced *Wolbachia* can cross several tissue planes and infect the germline in *Drosophila* [34]. This could be also the case in filarial *Wolbachia*, and it is possible that similar host signals trigger the germline tropism of *Wolbachia* in filarial worms and *Drosophila*. Previous studies have shown that a *Wolbachia* htrA serine protease can be found outside bacterial cells in filarial parasites. This protease and other secreted bacterial proteins may be involved in tissue invasion [35]. In addition to tissue lysis, motility of *Wolbachia* may be necessary for the bacteria to cross tissue boundaries. Actin-based motility occurs in *Rickettsia* and many other intracellular bacteria [36]. Orthologs of genes essential for actin-based motility have been found in the *Wolbachia* genome. Additional work will be needed to study the localization and timing of expression for these genes [23], [37].

Our ultrastructural results confirmed the presence of large clusters of *Wolbachia* in the lateral chords in the vicinity of the ovaries and in the outer ovary epithelium as previously described [30], [38]. The new finding reported here, is the detection of extracellular *Wolbachia* in the pseudocoelomic cavity in young females and the presence of *Wolbachia* in testis of developing male worms. In summary, this study shows the value of histological techniques such as immunohistology and *in situ* hybridization to study the tissue distribution of *Wolbachia* during the life cycle of filarial nematodes. *Wolbachia* infection was found to be highly cell and tissue specific. No *Wolbachia* were found in the developing reproductive organs in fourth stage larvae and freshly molted adult worms, which had heavy *Wolbachia* loads in the lateral chords. *Wolbachia* were detected in reproductive tissues with the onset of oocyte and sperm development, and infection of oocytes results in transovarial transmission of *Wolbachia* to the next generation.

## Supporting Information

Table S1 **Summary table for the parasite material used for the present study.** Each slide was thoroughly examined and numerous pictures were taken. If a slide contained more than one block section, all sections were analyzed.
(RTF)

Video S1 **Full rotation of a cross-section of a 5 week old female** ***B. malayi.*** *Wolbachia* were detected in the hypodermal lateral chords and the hypodermis. Confocal laser scanning microscopy stained with mab *Bm* WSP and WGA 633 (red) to visualize membranes. Compare Fig. 6A.
(MP4)

Video S2 **Similar to video S1 but** ***Wolbachia*** **are already attached to the membrane of the one ovary branch.** Note these attached bacteria can be noticed only from one side of the section. A few additional endobacteria are already in the ovary. The second ovary branch is still devoid of *Wolbachia*. Compare Fig. 6B.
(MP4)

Video S3 **Longitudinal section of a 5 week old female** ***B. malayi.*** *Wolbachia* are lining up at the lateral chords and some *Wolbachia* in one ovary branch. Confocal laser scanning microscopy stained with mab *Bm* WSP and WGA 633 (red) to visualize membranes. Compare Fig. 6C.
(MP4)
