# Supplementary material for: Tissue and Stage-Specific Distribution of Wolbachia in Brugia malayi
Source: PLoS Negl Trop Dis. 2011 May 24;5(5):e1174. doi: 10.1371/journal.pntd.0001174 (PMC3101188; doi:10.1371/journal.pntd.0001174)
Supplement: Table S1 — Summary table for the parasite material used for the present study. Each slide was thoroughly examined and numerous pictures were taken. If a slide contained more than one block section, all sections were analyzed. (RTF) [file pntd.0001174.s001.rtf]

Supplementary table: Overview about the B. malayi material examined in the present study. 
Stage	No parasites examined	Fixation (no. of blocks examined)	Method (no. of sections examined)	
Mf1	>500	Ethanol (5), formalin (3)	IH-A (4), IH-F (3), ISH (3), DAPI (3)	
L22	~25	Ethanol (5)	IH-A (5), IH-F (3), FISH (3), DAPI (4)	
L33	>500	Ethanol (5), formalin (3)	IH-A (5),  IH-F (5), FISH (3), ISH (3), DAPI (6)	
L4	~50	Ethanol (2), formalin (3)	IH-A (4), IH-F (4), FISH (3), ISH (3), DAPI (5) 	
Female 5 wks	46	Ethanol (3), formalin (3), glutaraldehyde-TEM (3) 	IH-A (9), IH-F (6), FISH (6), ISH (6), DAPI (6), TEM (5)	
Male 5 wks	23	Ethanol (2), formalin (2), glutaraldehyde-TEM (3)	IH-A (6), IH-F(4), FISH (4), ISH (4), DAPI (4), TEM (5)	
Female 8 wks	24	Formalin (4), glutaraldehyde-TEM (2)	IH-A (4), IH-F (4), FISH (3), ISH (3), DAPI (5), TEM (3)	
Male 8 wks	20	Formalin (4), glutaraldehyde-TEM (2)	IH-A (4), IH-F (4), FISH (3), ISH (3), DAPI (6), TEM (3)	
Female 12 wks	30	Ethanol (5), formalin (4), paraformaldehyde (1)	IH-A (7), IH-F (4), FISH (3), ISH (3), DAPI (4)	
Male 12 wks	28	Ethanol (4), formalin (3), paraformaldehyde (1)	IH-A (7), IH-F (4), FISH (3), ISH (3), DAPI (4) 	
1Stretched intrauterine mf or isolated mf (1 block); 2L2 in mosquitoes; 3L3 in mosquitoes or isolated L3 (2 blocks).  Each section (slide) contained multiple cross-, longitudinal- and saggital-sections of identical or different parasites.  IH-A, immunohistology (APAAP method) using mab Bm WSP as primary antibody; IH-F, immunohistology (immune fluorescence method) using mab Bm WSP as primary antibody; FISH, fluorescence in situ hybridization using oligonucleotides targeting the Wolbachia 16S rRNA; ISH, in situ hybridization using a plasmid derived RNA probe targeting the Wolbachia 16S rRNA; DAPI, DAPI stain for condensed DNA (often combined with other fluorescence-based staining); TEM, transmission electron microscopy.
